# Transcriptome sequencing and functional verification revealed the roles of exogenous magnesium in tobacco anti-PVY infection

**DOI:** 10.3389/fmicb.2023.1232279

**Published:** 2023-07-27

**Authors:** Huiyan Guo, Chuantao Xu, Fei Wang, Lianqiang Jiang, Xiao Lei, Mingjin Zhang, Rui Li, Xinyu Lan, Zihao Xia, Zhiping Wang, Yuanhua Wu

**Affiliations:** ^1^Liaoning Key Laboratory of Plant Pathology, College of Plant Protection, Shenyang Agricultural University, Shenyang, China; ^2^Luzhou Branch of Sichuan Province Tobacco Company, Luzhou, China; ^3^Liangshan Branch of Sichuan Province Tobacco Company, Xichang, China

**Keywords:** PVY, Mg, transcriptome, virus-induced gene silencing, resistance

## Abstract

Potato virus Y (PVY) infection causes necrosis and curling of leaves, which seriously affect the yield and quality of Solanaceous crops. The roles of nutrient elements in the regulation of plant resistance to virus infection has been widely reported, while the mechanisms are poorly studied. Previous studies in our laboratory have demonstrated that foliar spraying of MgSO_4_ could induce *Nicotiana tabacum* resistance to PVY by increasing the activity of defense-related enzymes. Consistent with the results, we found that exogenous magnesium (Mg) had a certain effect on *N. tabacum* anti-PVY infection. Meanwhile, Illumina RNA sequencing revealed that Mg induced resistance to PVY infection was mainly by regulating carbohydrate metabolism and transportation, nitrogen metabolism, Ca^2+^ signal transduction and oxidative phosphorylation. Moreover, we used virus-induced gene silencing assays to verify the function of homologs of five *N. tabacum* genes involved in above pathways in *N. benthamiana*. The results showed that *NbTPS* and *NbGBE* were conducive to PVY infection, while *NbPPases* and *NbNR* were related to resistance to PVY infection. These results suggested a novel strategy for resistance to PVY infection and provided a theoretical basis for virus-resistance breeding.

## 1. Introduction

Potato virus Y (PVY) belongs to the genus *Potyvirus* in the family *Potyviridae* (Rybicki, 2015). It causes serious economic losses in Solanaceous crops worldwide (Scholthof et al., 2011). Existing studies have mainly classified PVY strains into common strain (PVY^O^), tobacco veinal necrosis strain (PVY^N^) and stipple streak strain (PVY^C^) (Ellis et al., 1997). PVY^N^ infection usually induces tobacco vein necrosis (TVN) symptom, and often causes synergistic infection with other viruses, i. e. potato virus X (PVX), cucumber mosaic virus (CMV) and tobacco mosaic virus (TMV) (Karasev et al., 2011). In recent years, specific real-time reverse transcriptase-polymerase chain reaction (RT-PCR) assays and high-throughput sequencing methods have been greatly improved, which is helpful for rapid diagnosis of plant virus disease (Balme-Sinibaldi et al., 2006; Nie and Singh, 2022). However, the control of PVY is still difficult due to complex strain types and variation (Green et al., 2018). Screening host resistance genes and breeding resistant varieties are still the most fundamental approach to prevent PVY infection. The research of PVY-tobacco plants interactions proved that translationally controlled tumor protein (*NtTCTP*) mRNA was targeted by PVY virus-derived small interfering RNAs (vsiRNAs), and associated with PVY resistance (Guo et al., 2017). Eukaryotic translation initiation factors are closely related to viral replication, and inhibition of *eIF4E1-S* and *eIF (iso)4E-T* expression in tobacco can improve resistance to PVY (Le et al., 2022). These important host genes provide genetic resources for future PVY-resistant crop breeding.

Magnesium (Mg) is one of the essential nutrients for plants (Shaul, 2002). In the process of plant growth and development, Mg participates in plant photosynthesis, regulates chlorophyll synthesis, and is closely related to signal transduction and energy metabolism (Waters, 2011; Kleczkowski and Igamberdiev, 2021). Mg uptake by plants is affected by soil pH, texture and environmental conditions (Farhat et al., 2015). Oxidative damage caused by Mg deficiency usually leads to dwarfing of seedlings and interveinal chlorosis on older leaves (Cakmak and Yazici, 2010). Many studies have shown that exogenous Mg plays an important role in plant response to biotic and abiotic stresses. The growth activity and oxidative stress tolerance of tobacco plants treated with Mg oxide nanoparticles (MgONPs) were increased (Cai et al., 2018). Mg^2+^ transporter genes, *GmMGT4* and *GmMGT5* can regulate plasmodesmata permeability to promote exchange between carbon (C) and nitrogen (N) in the nodule and contribute to the vegetative growth of soybean (Cao et al., 2022). Overexpression of the *Arabidopsis* high-affinity Mg^2+^ transporter gene *AtMGT1* can improve Mg uptake to increase aluminium (Al) toxicity tolerance in *Nicotiana benthamiana* (Deng et al., 2006). Mg is also a key factor in the activation of many enzymes, and is closely related to the regulation of many hormones (de Bang et al., 2021). Studies have shown that MgO pretreatment on tomato plants can activate the jasmonate (JA) signaling pathway and upregulate the expression of defense gene *MYELOCYTOMATOSIS ONCOGENE HOMOLOG 2* (*MYC2*), which contributes to the control of *Fusarium oxysporum* f. sp. *lycopersici* (Fujikawa et al., 2021). Foliar application of magnesium carbonate (MgCO_3_) results in upregulation of defense-related genes in grapevines, such as *β*-1,3-glucanase (*GLU*) and pathogenesis-related-protein 1 (*PR1*), which increases the resistance to downy mildew (El-Sharkawy et al., 2022). In addition, the positive effects of some nutrient elements, such as boron and iron, on plant response to viral infection have been widely reported (Bi et al., 2022; Guo et al., 2022). Therefore, it is of great significance to deeply and widely explore the roles of Mg in the process of plant disease resistance.

In this study, we found that Mg plays an important role in the resistance of *N. tabacum* to PVY infection. Illumina RNA sequencing (RNA-seq) of *N. tabacum* under four different treatments (PBS solution + H_2_O or P + H; PBS solution + magnesium or P + Mg; PVY + H_2_O or PVY + H; PVY + magnesium or PVY + Mg) at three time points, was performed to screen the genes responsible for PVY resistance under Mg application. In addition, virus-induced gene silencing (VIGS) was used to verify the function of homologs of five *N. tabacum* differentially expressed genes (DEGs) selected from several key pathways. Our results contribute to understanding of the molecular mechanisms underlying Mg-mediated resistance to PVY infection and provide candidate genes for PVY-resistance breeding.

## 2. Materials and methods

### 2.1. Plant growth and virus inoculation

*Nicotiana tabacum* L. cv. K326 and *N. benthamiana* plants were grown in the artificial climate chamber that maintained at 25°C (day/night), 16 h/8 h (light/dark) cycles and 65% relative humidity. Potato virus Y (PVY-LN, GenBank ID: JQ971975) was isolated and purified by our laboratory and propagated on tobacco. Tobacco leaves were sprayed twice with H_2_O or MgSO_4_ with concentration of 240 mg·L^−1^ at 4–5 leaf stage at 72 and 24 h before phosphate-buffered saline (PBS) solution or PVY inoculation, respectively (PBS solution + H_2_O or P + H; PBS solution + magnesium or P + Mg; PVY + H_2_O or PVY + H; PVY + magnesium or PVY + Mg). Crude extracts from 1 g of PVY-infected tobacco leaf tissues homogenized with 0.01 mol·L^−1^ PBS (pH = 7.2) was mechanically inoculated on the surface of tobacco leaves. The inoculated leaves of tobacco at 1 day post inoculation (dpi) and the systemic leaves of tobacco at 3 dpi, 5 dpi, 7 dpi and 9 dpi were harvested for measurement of virus accumulations. The inoculated leaves at 1 dpi and the systemic leaves of tobacco at 3 and 9 dpi were harvested for RNA-seq analysis. Each treatment was performed for three biological replicates with at least nine plants.

### 2.2. RNA-seq analyses

About 1 μg of total RNA from each sample was used as input for RNA-seq. The libraries were generated using a NEB Next Ultra RNA Library Prep Kit. The libraries were sequenced on an Illumina HiSeq TM2500 (Biomarker Technologies Co. Ltd., Beijing, China). The clean reads were mapped to the reference genome of *N. tabacum*.^1^ The relative gene expression levels were normalized as fragments per kilobase of transcript per million mapped reads (FPKM). We set the threshold of false discovery rate (FDR) < 0.05 and |log2 fold change| ≥ 1 as DEGs. Gene Ontology (GO) enrichment of DEGs were analyzed by a GOseq R packages based Wallenius non-central hyper-geometric distribution (Young et al., 2011). We used KOBAS software to perform KEGG analyses of DEGs (Mao et al., 2005). The sequencing data were deposited in the SRA database at NCBI with the accession number PRJNA903693.

### 2.3. WGCNA analyses

Weighted gene co-expression network analysis (WGCNA) was used to construct gene co-expression networks (Langfelder et al., 2009). Highly co-expressed gene modules were obtained using the WGCNA v3.1.1 package in R language (Langfelder et al., 2009). A gene expression adjacency matrix was constructed to analyze the network topology with an unsigned type of topological overlap matrix (TOM), a power β of 6, a minModuleSize of 15, and minimum height for merging modules of 0.08036.

### 2.4. Virus-induced gene silencing (VIGS) assays

We used a previously reported tobacco rattle virus (TRV) vector for virus-induced gene silencing (VIGS) assays (Bachan and Dinesh-Kumar, 2012). The constructions of TRV-based vectors were performed according to Guo et al. (2022). The primers used are shown in Supplementary Table 1. The TRV1 and TRV2 plasmids were transformed individually into *Agrobacterium tumefaciens* strain GV3101. Agrobacterium cultures carrying TRV1 vector and TRV2 vector were mixed equally in volume with each final concentration of OD_600_ = 0.5, which were then infiltrated into the fifth and sixth leaves of eight-leaf *N. benthamiana* plants. The upper two non-infiltrated *N. benthamiana* leaves were mechanically inoculated with PVY or PBS solution after 10 days post infiltration with TRV. The upper two systemically infected leaves were collected at 10 days post PVY inoculation.

### 2.5. Real-time quantitative PCR

Total RNA of tobacco K326 and *N. benthamiana* leaf tissues were extracted using TRIzol reagent (TIANGEN, Beijing, China). The first-strand cDNA was synthesized using 2 μg of total RNA. The real time quantitative PCR (RT-qPCR) was performed as previously reported (Guo et al., 2022). The expression level of *N. tabacum Ntubc2* (AB026056.1) and *N. benthamiana NbActin* (AY179605.1) was used as an internal control, respectively. The specific primers used in RT-qPCR detection are listed in Supplementary Table 1. All the experiments were performed with at least three independent biological replicates.

### 2.6. Western blotting

Total proteins of tobacco K326 and *N. benthamiana* were extracted using a Plant Protein Extraction Kit (Solarbio, Shanghai, China). The proteins separated by 12% SDS-PAGE electrophoresis and were transferred to 0.20 μm polyvinylidene fluoride (PVDF) membranes (Sangon Biotech, Shanghai, China), which were then incubated in blocking solution for 1 h (Solarbio, Shanghai, China). PVY CP antibody was used at a dilution of 1: 1000 (Youlong, Shanghai, China). Beta-actin antibody was used at a dilution of 1: 5000 (Proteintech, Chicago, USA). Secondary antibody was used at a dilution of 1: 10000 (ABclonal, Wuhan, China). After incubation with primary and secondary antibodies, membranes were washed twice for 15 min with 1 × TTBS. Finally, the membranes were transferred into ECL solution (Millipore, Billerica, USA) to detect signals by Tanon Chemiluminescence Gel Imager (Tanon, Shanghai, China).

### 2.7. Statistical analyses

IBM SPSS Statistics 25.0 software (IBM Inc., Armonk, USA) was used for data analysis. The differences among groups were analyzed through two-tailed *t* test and one-way analysis of variance (Duncan).

## 3. Results

### 3.1. Foliar application of Mg alleviated PVY infection in tobacco

**Figure 1:**
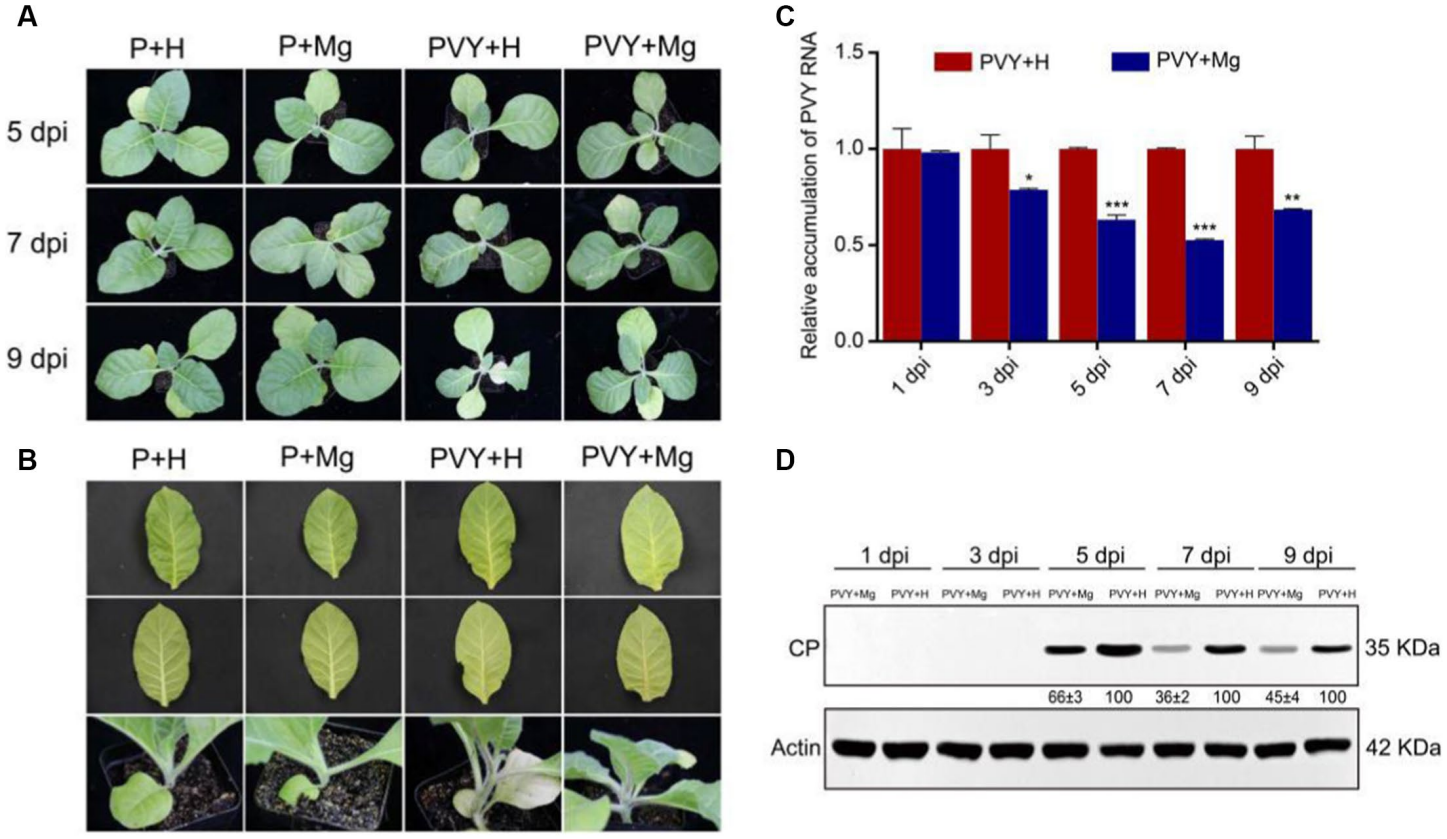
Symptoms and viral accumulation in tobacco after PVY infection under different treatments. **(A)** Symptoms of tobacco in four groups at 5, 7 and 9 dpi. **(B)** Close-up views of stems and leaves of tobacco in four groups at 9 dpi. **(C)** PVY genomic RNA accumulations determined by RT-qPCR in tobacco under different treatments. Asterisks indicate statistical difference between treatments, determined by the two-tailed *t* test (*p < 0.05, **p < 0.01, ***p < 0.001). **(D)** The accumulations of PVY CP proteins in PVY + Mg and PVY + H tobacco leaves at 1, 3, 5, 7, 9 dpi.

To verify the effect of Mg on tobacco anti-PVY infection, we set up four treatments (PBS solution + H_2_O or P + H; PBS solution + magnesium or P + Mg; PVY + H_2_O or PVY + H; PVY + magnesium or PVY + Mg) at five different time points. The results showed that milder PVY symptoms occurred on PVY + Mg plants compared with that on PVY + H plants (Figures 1A,B). At 9 dpi, the PVY + H plants showed severe stems necrosis and leaves chlorosis, while those PVY + Mg plants showed only slight stem browning (Figures 1A,B). RT-qPCR and Western blot were used to detect the accumulations of genomic RNAs and CP proteins of PVY in the upper two tobacco leaves, and the results were consistent with the severity of our observed symptoms (Figures 1C,D). At 7 dpi, the accumulations of PVY genomic RNAs and CP proteins were decreased by 48 and 64% in PVY + Mg plants, compared with that in PVY + H plants (Figures 1C,D). These results indicated the positive effect of Mg on inhibiting PVY infection in tobacco.

### 3.2. Illumina RNA sequencing

In order to explore the molecular mechanism of Mg regulation on tobacco resistance to PVY infection, we performed four different treatments on tobacco plants at 1, 3, 9 dpi for Illumina RNA-seq, resulting in a total of 36 libraries. The correlation analysis of three replications of 36 samples showed that Pearson’s correlation coefficients were between 0.586 and 0.995, which proved that the correlation between each group of biological replicates was high (Supplementary Figure 1). Each library contained ≥6.11 Gb of clean data with Q30 quality scores ≥92.98%, and CG content percentage between 43.36 and 45.11% (Supplementary Table 2). Mapped the sequencing reads to reference genome of *N. tabacum* cv. K326, the comparison efficiency ranged from 78.63 to 96.23% (Supplementary Table 3).

### 3.3. Comparative analyses of DEGs

**Figure 2:**
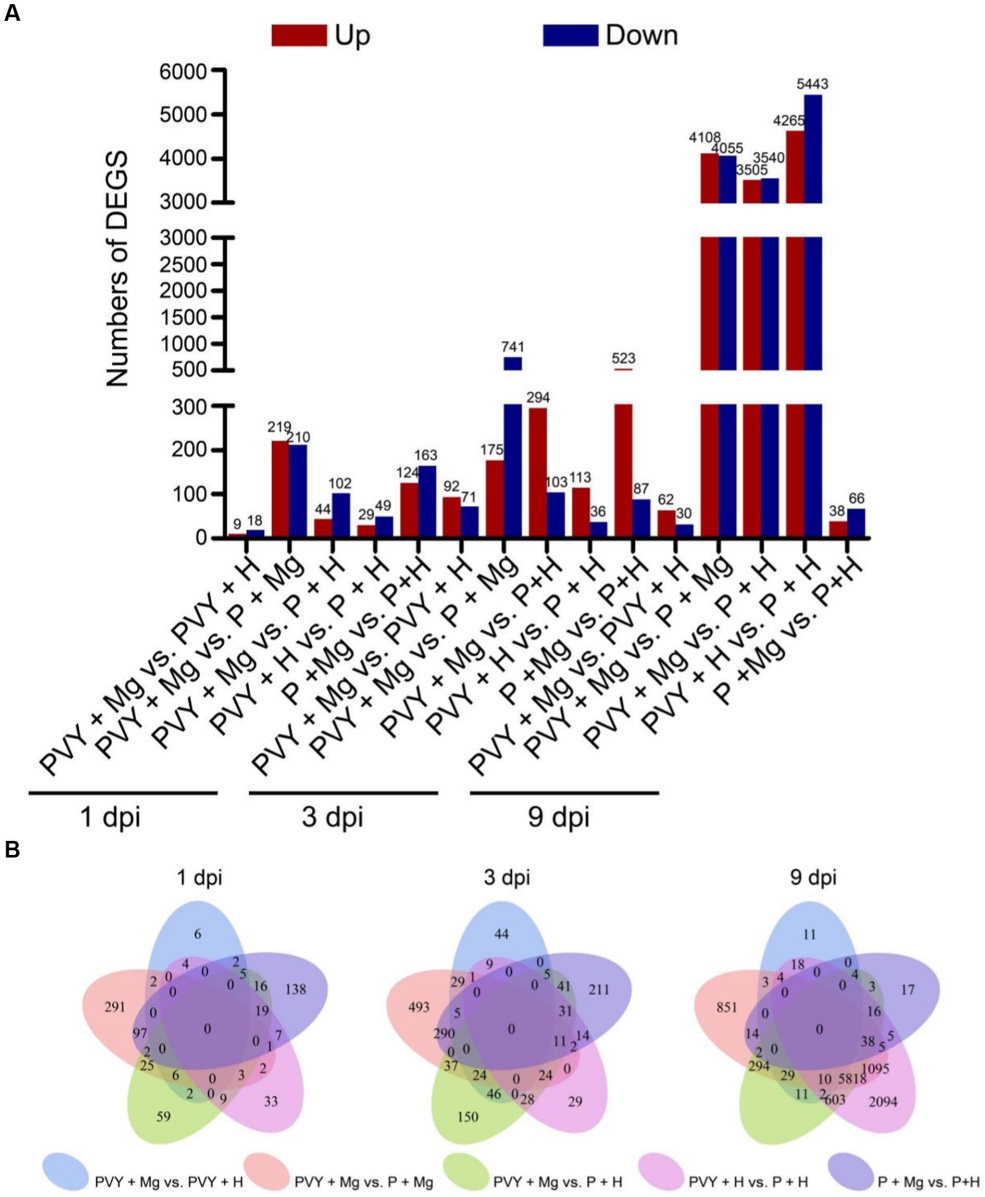
Analyses of differentially expressed genes (DEGs). **(A)** The up-and down-regulated DEGs in five comparisons at 1, 3, 9 dpi. **(B)** Venn diagrams of DEGs in five comparisons at 1, 3, 9 dpi.

To further elucidate the transcriptomic variations of tobacco leaves resistant to PVY infection induced by Mg application, we conducted pairwise comparison of different treatments (i.e., PVY + Mg vs. P + Mg, PVY + Mg vs. PVY + H, P + Mg vs. P + H, PVY + H vs. P + H and PVY + Mg vs. P + H) at 1, 3, 9 dpi. Based on the standard, a total of 11,970 DEGs were identified, of which 429, 27, 287, 78, and 146 DEGs were found in PVY + Mg vs. P + Mg, PVY + Mg vs. PVY + H, P + Mg vs. P + H, PVY + H vs. P + H and PVY + Mg vs. P + H at 1 dpi, respectively (Figures 2A,B). At 3 and 9 dpi, 163 and 92 DEGs were found in PVY + Mg vs. PVY + H (Figures 2A,B). The expressions of a total of 9,708 genes were changed in the PVY + H vs. P + H comparison at 9 dpi, significantly more than that at 1 and 3 dpi (Figures 2A,B). In P + Mg vs. P + H comparison, we found that the DEGs were mainly concentrated in 1 and 3 dpi (Figures 2A,B). In addition, more DEGs in PVY + Mg vs. PVY + H comparison at 3 dpi than that at 1 and 9 dpi (Figures 2A,B). These results indicated that the changes of gene expression in tobacco plants induced by PVY infection were increased with time, while the regulation of Mg on tobacco gene expression was mainly concentrated at 3 dpi during PVY infection.

### 3.4. GO and KEGG enrichment analyses of DEGs

**Figure 3:**
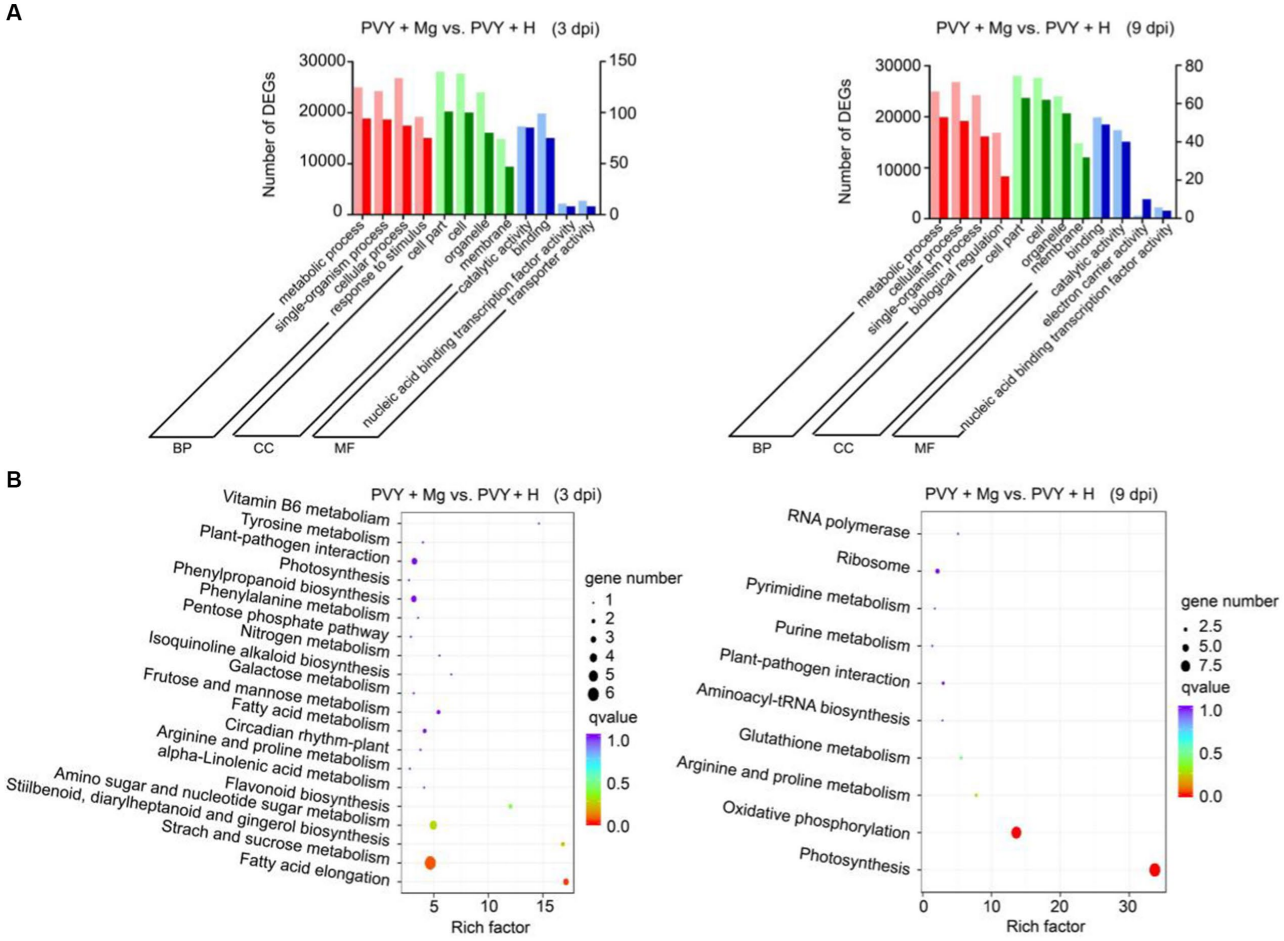
Enrichment analyses of DEGs. **(A)** GO analyses of DEGs in PVY + Mg vs. PVY + H at 3 and 9 dpi. (MF, molecular function; CC, cellular component; BP, biological process). **(B)** KEGG analyses of DEGs in PVY + Mg vs. PVY + H at 3 and 9 dpi.

To further explore the effects of PVY infection on tobacco under Mg treatment, we selected DEGs in PVY + Mg vs. PVY + H at 3 and 9 dpi for GO and KEGG pathway enrichment analyses, respectively. The GO terms in PVY + Mg vs. PVY + H at 3 dpi were mainly enriched in the biological process (BP) terms ‘metabolic process’ and ‘single-organism process’, cellular component (CC) terms ‘cell part’ and ‘cell’, and the molecular function (MF) terms ‘catalytic activity’ and ‘binding’ (Figure 3A; Supplementary Table 4). The results of GO analyses at 9 dpi were basically consistent with those at 3 dpi (Figure 3A; Supplementary Table 4). Subsequent KEGG enrichment analyses showed that DEGs in PVY + Mg vs. PVY + H at 3 dpi were significantly enriched in ‘starch and sucrose metabolism’ (ko00500), ‘fatty acid elongation’ (ko00062), ‘amino sugar and nucleotide sugar metabolism’ (ko00520) and ‘nitrogen metabolism’ (ko00910), while ‘photosynthesis’ (ko00195), ‘oxidative phosphorylation’ (ko00190) and ‘arginine and proline metabolism’ (ko00330) were the top three pathways at 9 dpi (Figure 3B).

### 3.5. WGCNA analyses of DEGs

**Figure 4:**
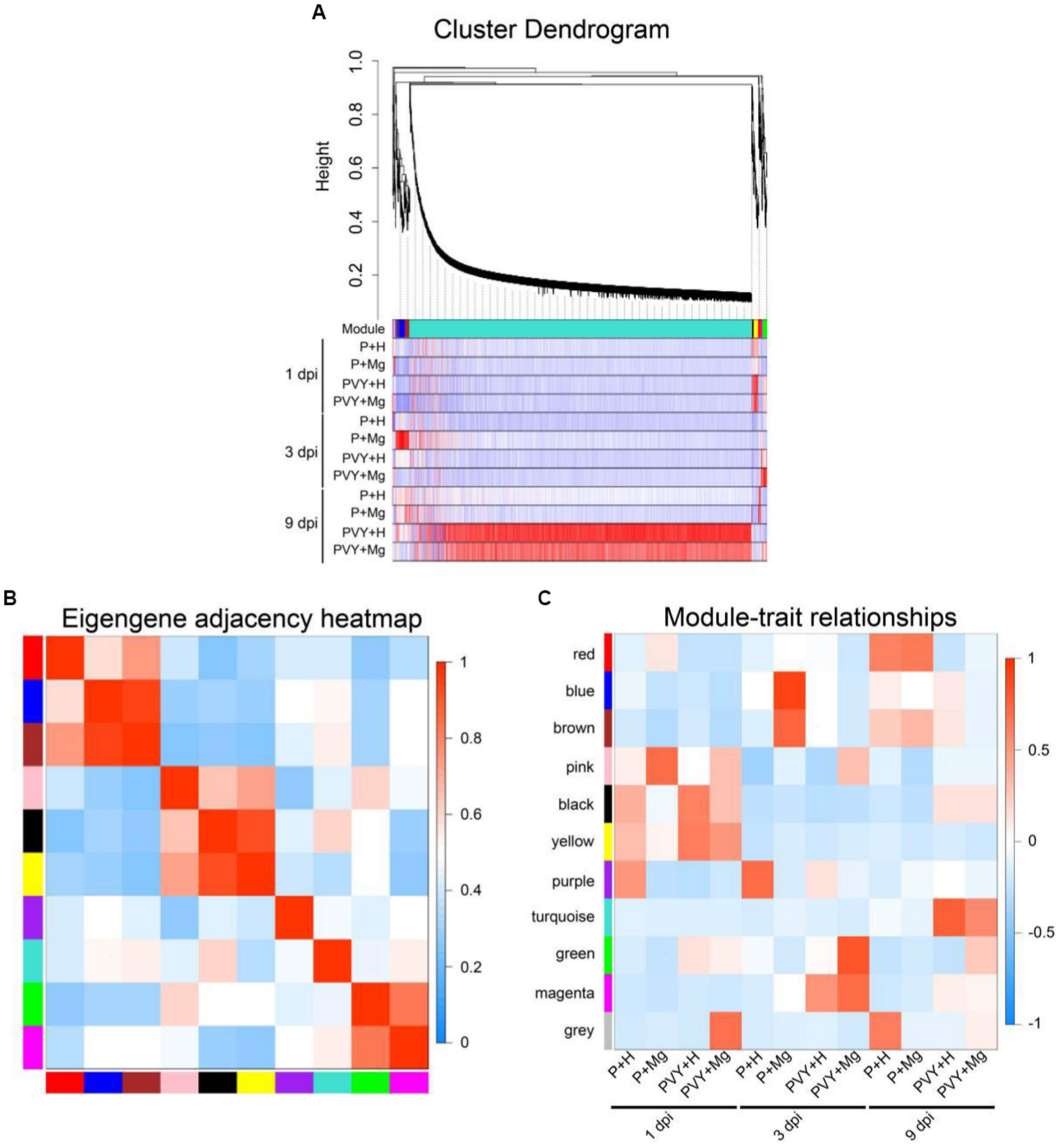
DEGs of WGCNA analyses. **(A)** Hierarchical cluster tree and heatmap of all DEGs. The hierarchical cluster tree shows co-expression modules identified through WGCNA. Each leaf in the tree represents one DEG. The major tree branches constitute four modules labelled with different colors. The heatmap shows the relative expressions of the whole DEGs in different modules. **(B)** Eigengene adjacency heatmap of the four modules shows the correlations among different modules. The darker red represents a higher correlation. **(C)** Associations between modules and traits. The colors of the modules are the same as that shown in panels **(A,B)**. The numbers in individual cells represent the correlations and the *p* values.

To further analyze the gene regulatory network of Mg regulating resistance to PVY infection in tobacco, we performed WGCNA analysis on all the obtained genes. We finally identified 10 different gene regulatory modules containing DEGs ranging from 19 to 4006 (Figure 4A; Supplementary Table 5). The correlations between modules and modules, and modules and treatments were showed (Figures 4B,C). The results showed a strong correlation between green and magenta modules and these DEGs were highly expressed in PVY + Mg at 3 dpi. Top GO and KEGG analyses showed that these DEGs were mainly enriched in ‘energy production and conversion’, ‘photosynthesis’, ‘plant-pathogen interaction’ and ‘protein processing in endoplasmic reticulum’. These two modules mainly included DEGs related to oxidative phosphorylation (*NtAS*, *NtNQOR*), photosystem II (*NtPsbC*), photosystem I (*NtPSI-A2*) and some chaperones (*NtHSP90*, *NtHSP20*). The turquoise and black modules were highly correlated with PVY + Mg at 9 dpi. These DEGs were mainly enriched in ‘inorganic ion transport and metabolism’, ‘carbohydrate transport and metabolism’ and ‘plant-pathogen interaction and respiratory burst’, which were mainly involved in starch and sucrose metabolism (*NtSS*, *NtTPS*), nitrogen metabolism (*NtrTl-Cyn*, *NtNRT*), Ca^2+^ signal transduction (*NtCML44*, *NtCML35*, *NtCML41*, *NtCML36*) and ROS scavenging (*NtPrx*) (Supplementary Tables 6, 7). Through WGCNA analysis, we found that the DEGs in green and magenta modules were involved in the resistance of tobacco to PVY infection regulated by Mg at 3 dpi, while the DEGs in turquoise and black modules were involved in the resistance of tobacco regulated by Mg at 9 dpi.

### 3.6. Analyses of DEGs involved in carbohydrate transport and metabolism pathways

**Figure 5:**
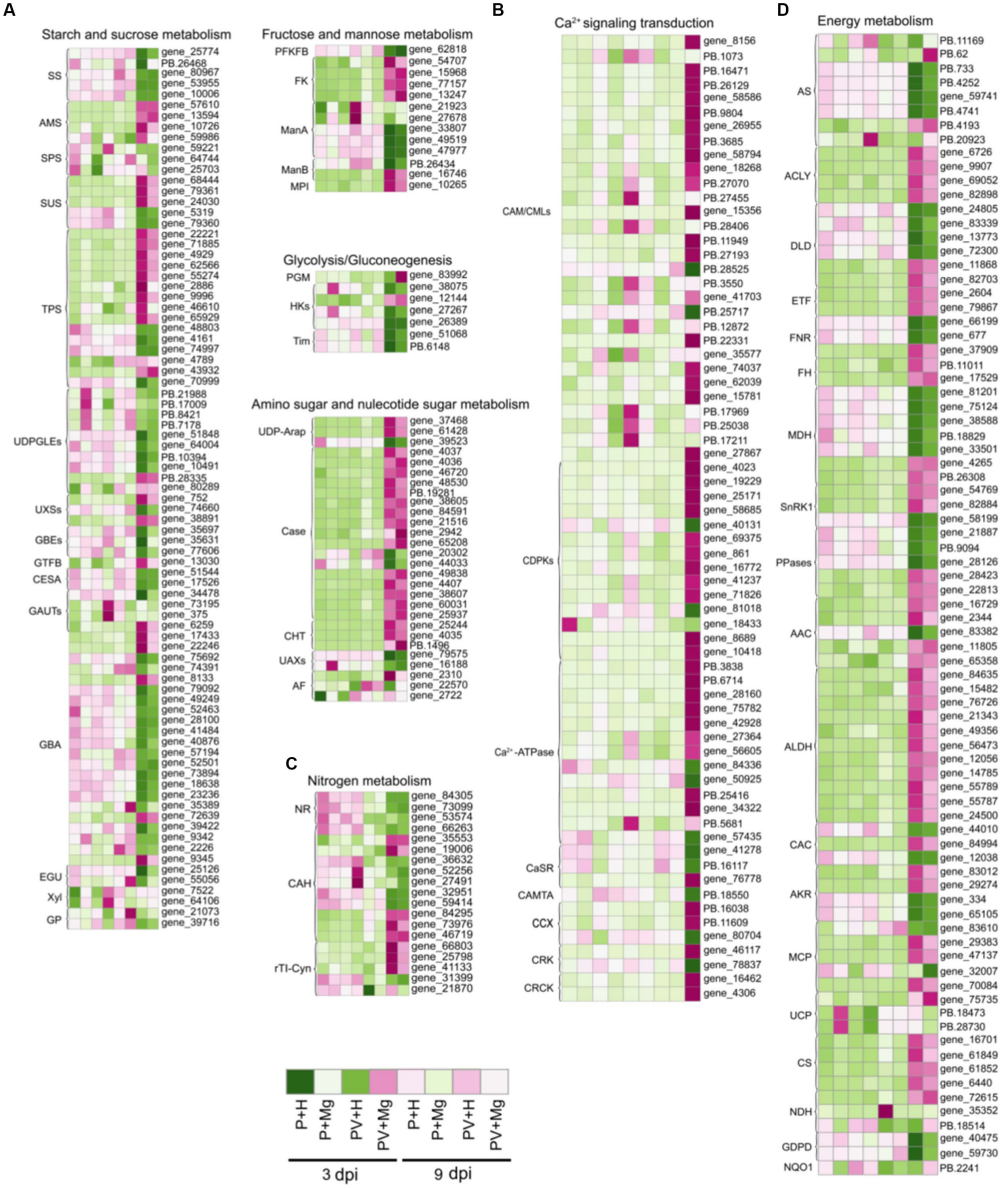
Heap map diagrams of relative expression levels of DEGs. **(A)** DEGs in carbohydrate transport and metabolism. **(B)** DEGs in Ca^2+^ signaling transduction. **(C)** DEGs in nitrogen metabolism. **(D)** DEGs in energy metabolism.

Carbohydrate metabolism is closely related to other metabolic activities, which provides energy for plant growth and enhances plant stress resistance (Pommerrenig et al., 2018). In starch and sucrose metabolism pathway, we found 83 DEGs at 1, 3 and 9 dpi under different treatments, including five starch synthases (*NtSSs*), four amylases (*NtAMSs*), three sucrose-phosphate synthases (*NtSPSs*), five sucrose synthases (*NtSUSs*), 15 alpha, alpha-trehalose-phosphate synthases (*NtTPSs*), 10 UDP-glucuronate 4-epimerases (*NtUDPGLEs*), three UDP-glucuronic acid decarboxylases (*NtUXSs*), three 1,4-alpha-glucan-branching enzymes (*NtGBEs*), one 4-alpha-glucanotransferase (*NtGTFB*), two cellulose synthase-like proteins (*NtGESAs*), four galacturonosyltransferases (*NtGAUTs*), 22 glucosidases (*NtGBAs*), two endoglucanases (*NtEGUs*), two beta-D-xylosidases (*NtXyls*) and two alpha-1,4 glucan phosphorylase L-2 isozymes (*NtGPs*) (Figure 5A; Supplementary Table 8). At 9 dpi, the expression levels of almost all these DEGs were up-or down-regulated in PVY + H vs. P + H. In PVY + Mg vs. PVY + H, the expression level of *NtSPSs*, *NtGAUT12*, *NtXyl5* were up-regulated at 3 dpi, and *NtGBEs* and *NtTPSs* were down-regulated to some extent at 9 dpi. We identified 13 DEGs associated with fructose and mannose metabolism pathway, of which fructokinase-1 isoform X1 (*NtFKs*) and mannose-6-phosphate isomerase 1-like (*NtMPIs*) were up-regulated in PVY + H vs. P + H at 9 dpi, while mannan endo-1,4-beta-mannosidases (*NtManAs*) were down-regulated (Figure 5A; Supplementary Table 8). In PVY + Mg vs. PVY + H, we found that *NtManAs* were up-regulated at 3 dpi. In glycolysis / gluconeogenesis pathway, seven DEGs were obtained, including one phosphoglucomutase (*NtPGM*), four hexokinases (*NtHKs*) and two triosephosphate isomerases (*NtTims*) (Figure 5A; Supplementary Table 8). Most of these DEGs were down-regulated in PVY + H vs. P + H at 9 dpi, while unchanged in PVY + Mg vs. PVY + H. There were 28 DEGs related to amino sugar and nucleotide sugar metabolism pathway (Figure 5A; Supplementary Table 8). In PVY + H vs. P + H, UDP-arabinose 4-epimerases (*NtUDP-Araps*), chitinases (*NtCases*), most of endochitinases (*NtEases*) and chitotriosidases (*NtCHTs*) were up-regulated at 9 dpi, while UDP-D-apiose/UDP-D-xylose synthases (*NtUAXs*) were down-regulated. These results suggested that PVY infection seriously affected plant carbohydrate metabolism at 9 dpi, while mannose metabolism and sucrose metabolism were regulated by Mg under PVY infection.

**Figure 6:**
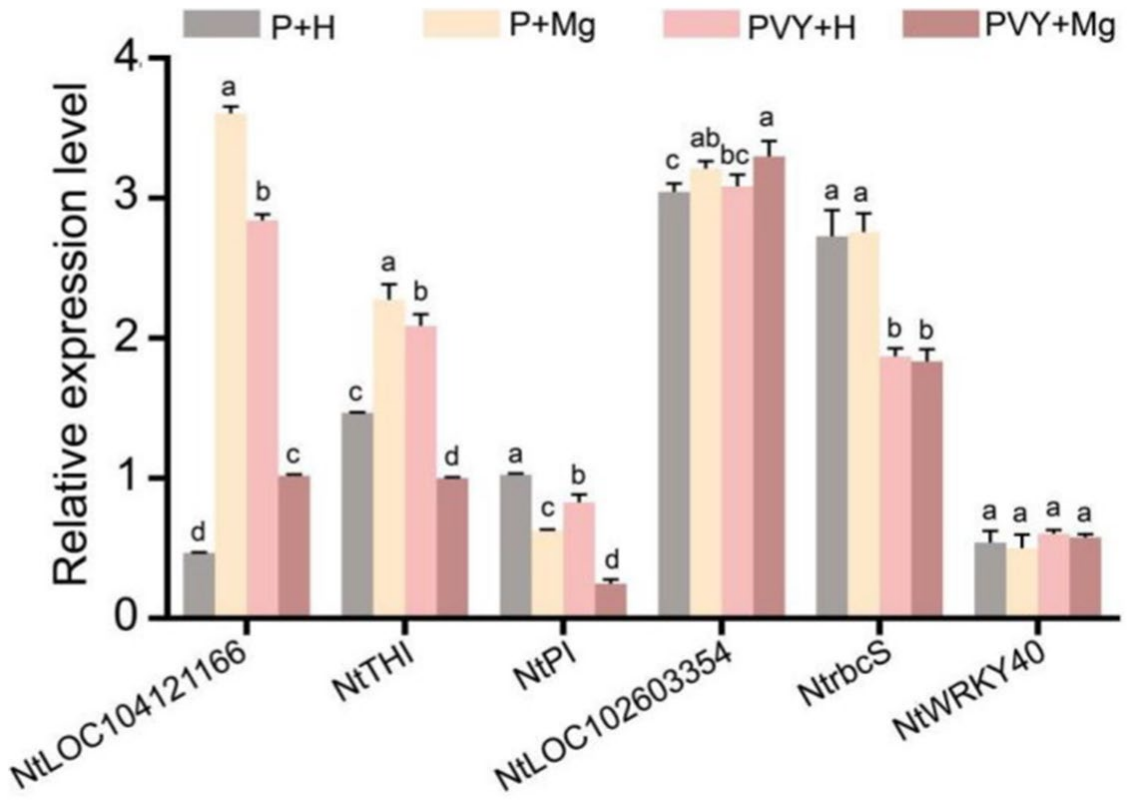
The expression levels of four genes determined by RT-qPCR under different treatments. Lowercase letters indicate statistical difference between treatments. The statistical significances were determined using one-way analysis of variance followed by Duncan’s multiple comparison test (*p* value <0.05).

### 3.7. Analyses of DEGs involved in Ca^2+^ signaling transduction

Reactive oxygen species (ROS) bursts and allergic reactions induced by Ca^2+^ concentration in intracellular and extracellular are important immune mechanisms in plant response to stresses (Moeder et al., 2019). In this study, we identified 68 DEGs in Ca^2+^ signaling transduction pathway at 9 dpi in PVY + H vs. P + H (Figure 5B; Supplementary Table 8), of which 35 DEGs were up-regulated including 14 *NtCAM*/*CMLs*, eight calcium-dependent protein kinases (*NtCDPKs*), nine calcium-transporting ATPases (*NtCa^2+^-pumps*), one calmodulin-binding transcription activator (*NtCAMTA*), one cation/calcium exchanger (*NtCCX*) and two calmodulin-binding receptor-like cytoplasmic kinases (*NtCRCKs*), while 13 DEGs were down-regulated including three *NtCAM*/*CMLs*, three *NtCDPKs*, two *NtCa^2+^-pumps*, three calcium sensing receptors (*NtCaSRs*), one *NtCCX* and one CDPK-related kinase (*NtCRK*), but no DEGs were found at 1 dpi and 3 dpi. In PVY + Mg vs. PVY + H, we found that only *NtCDPK29* was up-regulated at 3 dpi, while three *NtCAM/CMLs* and one *NtCa^2+^-ATPase* were down-regulated at 9 dpi. These results showed that Ca^2+^ signaling transduction was involved in response to PVY infection and Mg application may inhibit part of Ca^2+^ flow at 9 dpi.

### 3.8. Analyses of DEGs involved in nitrogen metabolism

Nitrogen is an essential nutrient for plant growth, and nitrogen metabolism is one of the important ways for plants to resist stresses (Wang et al., 2014). In this study, we found 19 DEGs involved in nitrogen metabolism (Figure 5C; Supplementary Table 8). At 9 dpi, one nitrite reductase (*NtNR*) and two carbonic anhydrases (*NtCAHs*) were down-regulated in PVY + H vs. P + H, while two cyanate hydratases (*NtrTI-Cyns*) were up-regulated. At 3 dpi, only one *NtCAH* was down-regulated in PVY + Mg vs. PVY + H.

### 3.9. Analyses of DEGs involved in oxidative phosphorylation pathways

Plant energy metabolism is the basis of life activities (Siqueira et al., 2018). By analyzing all DEGs at 1, 3 and 9 dpi, we identified a total of 80 DEGs related to oxidative phosphorylation (Figure 5D; Supplementary Table 8). At 9 dpi, most of the enzymes related to oxidative phosphorylation were up-or down-regulated to varying degrees (i. e. *NtASs*, *NtACLYs*, *NtDLDs*, *NtETFs*, *NtFNRs*, *NtFHs*, *NtMDHs*, *NtSnRK1s*, *NtPPases*, *NtAACs*, *NtALDHs*, *NtCACs*, *NtAKRs*, *NtMCPs*, *NtUCPs*, *NtCSs*, *NtNDHs* and *NtGDPDs*) in PVY + H vs. P + H comparison. At 1 and 3 dpi, however, the expression levels of these DEGs were unchanged. In PVY + Mg vs. PVY + H comparison, we found that three *NtASs* related to mitochondrial oxidative phosphorylation reaction, and one *NtNDH* related to respiratory chain were up-regulated at 9 dpi. These results indicated that PVY infection interfered with plant energy metabolism, and exogenous Mg could improve the activity of related enzymes to a certain extent, thus promoting the energy metabolism of plants.

### 3.10. Validation of RNA-seq data

We randomly selected six genes to verify their expression levels in four different treatments at three time points (Figure 6). RT-qPCR results showed that the expression levels of *LOC104121166* (gene_42165) and *NtTHI* (gene_395) were down-regulated in PVY + Mg vs. PVY + H, while up-regulated in PVY + H vs. P + H at 1 dpi. The expression levels of *NtrbcS* (gene_61355) were unchanged in PVY + Mg vs. PVY + H at 1 dpi, while down-regulated in PVY + H vs. P + H. The expression levels of *NtPI* (gene_80129) were down-regulated in PVY + Mg vs. PVY + H and PVY + H vs. P + H at 3 dpi. The expression levels of *NtWRKY40* (gene_48638) were unchanged in four different treatments at 3 dpi. The expression levels of *LOC102603354* (PB.15726) were unchanged in PVY + H vs. P + H, while up-regulated in PVY + Mg vs. PVY + H at 9 dpi. These results are basically consistent with our transcriptome sequencing results.

### 3.11. Functional validation of homologous genes of tobacco in response to PVY infection in *N. benthamiana*

**Figure 7:**
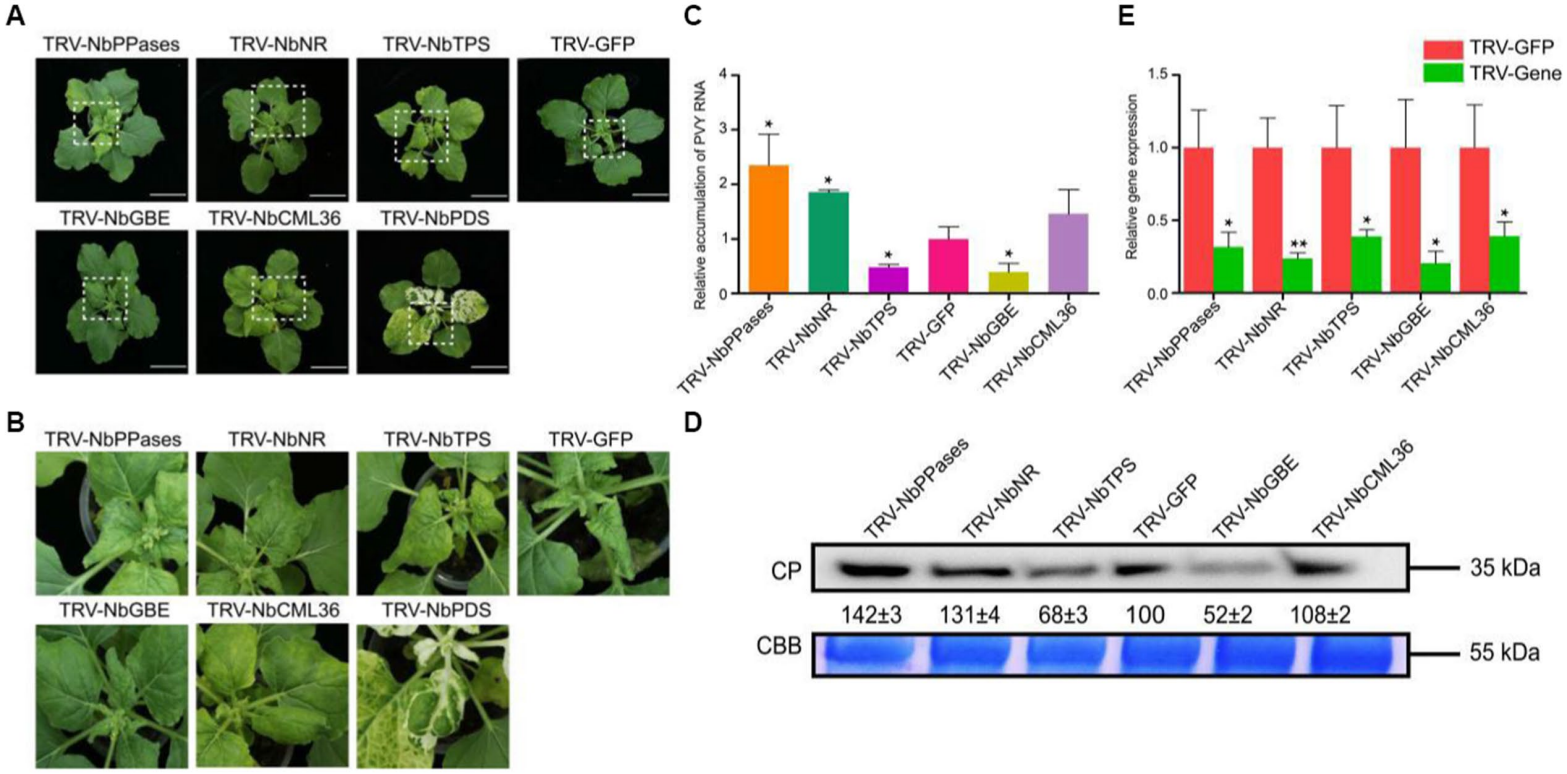
Functional validation of homologous DEGs of tobacco in response to PVY infection in *N. benthamiana*. **(A)** Disease symptoms on *NbGBE*-, *NbTPS*-, *NbPPases*-, *NbCML36-or NbNR*-silenced *N. benthamiana* after PVY infection. **(B)** Close-up views of upper leaves indicated by white dash boxes in **(A)**. **(C)** Relative accumulation of PVY RNA in *N. benthamiana* upper leaves. Asterisks indicate statistical difference between treatments, determined by the two-tailed *t* test (*p < 0.05). **(D)** Relative accumulation of PVY CP proteins in *N. benthamiana* upper leaves. **(E)** Silencing efficiencies of target genes determined through RT-qPCR. Asterisks indicate statistical difference between treatments, determined by the two-tailed *t* test (*p < 0.05, **p < 0.01).

Transcriptome sequencing results showed that *NtNR* and *NtPPases* that involved in nitrogen metabolism and energy metabolism were down-regulated in PVY + H vs. P + H at 9 dpi. *NtTPS* and *NtGBE* related to carbohydrate transport and metabolism were down-regulated in PVY + Mg vs. PVY + H at 9 dpi, while *NtCML36* related to Ca^2+^ signaling transduction was up-regulated. In order to further explore the roles of these five DEGs, we selected their homologous genes in *N. benthamiana* and performed functional analyses through TRV-based VIGS assays. At 10 dpi, we found that *NbGBE*-silenced *N. benthamiana* had lower PVY accumulation and weaker symptoms, while *NbNR-and NbPPases*-silenced plants accumulated higher PVY and showed more serious leaf curl and chlorosis (Figures 7A–D). The PVY accumulation in *NbTPS*-silenced plants was significantly reduced compared with that in control plants, and the severe yellowing symptoms of these plants may be caused by gene silencing combined with PVY infection (Figures 7A–D). The virus accumulation in *NbCML36*-silenced plants was almost the same as that in control plants (Figures 7A–D). The silencing efficiency of target genes ranged from 55 to 85% determined by RT-qPCR (Figure 7E).

## 4. Discussion

The necrosis in leaf veins and stems caused by PVY infection is an irreversible loss in production of Solanaceous crops (Quenouille et al., 2013). It has been reported that Mg plays an important role in plant photosynthesis and carbohydrate metabolism (Courtois et al., 2003). In this study, we found that application of exogenous Mg had a certain inhibitory effect on PVY infection (Figure 1). Through RNA-seq, we found carbohydrate metabolism, nitrogen metabolism and oxidative phosphorylation as the major anti-PVY infection pathways regulated by Mg (Figure 3). To validate this hypothesis, we selected five *N. tabacum* homologous genes in *N. benthamiana* for functional verification (Figure 7). Finally, the functions of these candidate genes in tobacco resistance to PVY infection were preliminarily clarified. However, the molecular mechanism is still needed to be investigated.

In plants, carbohydrates, including glucose, sucrose and fructose, account for a large proportion of all energy substance in plants (Julius et al., 2017). Pathogen infection affects photosynthesis and carbohydrate metabolism of host plants, leading to suppression of immune responses (Kanwar and Jha, 2019). Studies have shown that glucose can regulate phenylalanine ammonilyase activity to induce defense-related responses, and sucrose can also improve host resistance to *Fusarium oxysporum* by promoting isoflavonoid accumulation (Morkunas et al., 2005; Kim and Hwang, 2014). In watermelon plants, boron application can promote the resistance to CGMMV by increasing glucose and fructose content and decreasing sucrose content (Bi et al., 2022). In our study, a total of 107 DEGs of carbohydrate transport and metabolism pathways were identified in PVY + H vs. P + H at 9 dpi, suggesting that PVY infection significantly interfered with plant carbohydrate metabolism (Figure 5A; Supplementary Table 8). We found that some genes related to plant carbohydrate metabolism were activated by Mg at 3 dpi, and a total of six DEGs (i. e. *NtSPSs*, *NtGAUTs*, *NtXyls*, *NtManAs*) were found during this process. However, at 9 dpi, the expression of *NtTPSs* and *NtGBEs* were down-regulated in PVY + Mg vs. PVY + H. To further explore the gene function, we silenced the expression of the homologous genes of *NtTPS* and *NtGBE* using TRV-based VIGS in *N. benthamiana*. The leaves collected from the *NbTPS-and NbGBE*-silenced plants accumulated lower PVY genomic RNAs and CP proteins compared with the control groups (Figure 7). GBEs can catalyze the formation of new branches of glycogen and change the glycogen structure, thus favoring glycogen storage (Ban et al., 2020). Trehalose phosphate synthase is a key enzyme involved in trehalose synthesis pathway (Paul et al., 2008). Studies have shown that the ability of pathogens to infect hosts is related to their trehalose metabolism pathways. Knocking out *TPS1* gene of *Magnaporthe oryzae* showed significant lower trehalose synthesis and pathogenicity and the expression of virulence-associated genes was affected (Wilson et al., 2007). *Pseudomonas aeruginosa* mutants that unable to synthesize trehalose lost their infectivity to *Arabidopsis* (Djonović et al., 2013). Therefore, we surmised that exogenous application of Mg may inhibit PVY infection by regulating glycogen and trehalose metabolism.

Nitrogen metabolism is known to regulate plant stress resistance by stabilizing cellular structure and maintaining photosynthesis (Fagard et al., 2014). Studies on *Arabidopsis thaliana* with different resistant varieties showed that nitric oxide (NO) was an important signal of plant resistance to *Sclerotinia sclerotiorum* and correlated with the expression of defense-related genes (Perchepied et al., 2010). Conversely, some studies have reported the role of nitrogen transport metabolites in promoting pathogen infection, such as the transcriptional upregulation of asparagine synthetase in tomato infected with *Botrytis cinerea*, which provides a richer source of nitrogen for the pathogen and thus promotes the disease development (Seifi et al., 2014). However, the molecular mechanism of plant nitrogen metabolism participating in plant viral disease resistance has not been reported. In this study, we found that PVY infection affected tobacco nitrogen metabolism at 9 dpi (Figure 5B; Supplementary Table 8). In PVY + Mg vs. PVY + H, we found that *NtCAH* gene was down-regulated at 3 dpi. Carbonic anhydrase is closely related to nitrogen fixation in *Rhizobium* (Flemetakis et al., 2003). As a salicylic acid-binding protein SABP3, carbonic anhydrase plays an important role in plant immune response due to its antioxidant activity (Slaymaker et al., 2002). Nitrite reductase plays an important role in nitrate assimilation in plants (Costa-Broseta et al., 2020). In this study, we found that the accumulation of PVY in *NbNR*-silenced *N. benthamiana* was higher, which may be related to the disruption of plant nitrogen metabolism, thus affecting tobacco energy metabolism.

Elevated intracellular Ca^2+^ concentration is an early signal for plants to perceive pathogen invasion, which can induce the expression of defense-related response genes and increase plant stress resistance (Ma and Berkowitz, 2011). In this study, we found that the expression levels of most *CAM/CMLs* were changed in PVY + Mg vs. PVY + H and PVY + H vs. P + H comparisons at 9 dpi (Figure 5C; Supplementary Table 8). Overexpression of *CML43* in *Arabidopsis* can accelerate hypersensitive response (HR) (Chiasson et al., 2005). Overexpression of a pepper gene *CaCAM1* in Arabidopsis induced ROS burst, NO production and HR, and enhanced the resistance to *Pseudomonas syringae* (Choi et al., 2009). In tobacco, knocking down *NtCAM13* promoted pathogen infection, while silencing *NtCAM1* had no effect (Takabatake et al., 2007). This is similar to our VIGS results. We selected *NbCML36*, the homologous gene of tobacco *NtCML36*, for VIGS verification in *N. benthamiana*, and found that the virus accumulation in *NtCML36*-silenced plants increased slightly, but was not significant. Based on these above results, we can speculate that Mg affects ROS burst and HR in tobacco plants by regulating Ca^2+^ signal transduction, thus inhibiting PVY infection, yet *NbCML36* is not a major gene in tobacco response to PVY infection.

Oxidative phosphorylation (OXPHOS) is a key pathway for ATP production by mitochondria, which provides basic energy for cell life activities (Meyer et al., 2019). ATP synthetase is a key protease to maintain mitochondrial structure and function (Kühlbrandt, 2019). PVY HC-Pro interact with chloroplast ATP synthase NtCF1β-subunit, thus affect the assembly of the ATP synthase complex (Tu et al., 2015). MgADP is a substrate for ATP synthase, so the intracellular concentration of Mg^2+^ is closely related to the regulation of respiration (Gout et al., 2014). NADH dehydrogenase is the first proton pump in the electron transport chain, which can transfer electrons from NADH (Wirth et al., 2016). In this study, we found that three *NtASs* and one *NtNDH* genes were up-regulated in PVY + Mg vs. PVY + H (Figure 5D; Supplementary Table 8). Therefore, we hypothesized that Mg might enhance PVY resistance by promoting ATP production in tobacco. In addition, we also verified the function of *NbPPases* that is involved the hydrolysis of inorganic pyrophosphate, and the results showed that silencing *NbPPases* promoted the accumulation of PVY in *N. benthamiana*.

In summary, we confirmed that Mg application could effectively inhibit PVY infection in tobacco and demonstrated the pathways that were regulated by Mg under PVY infection by RNA-seq. Our findings provide a clear picture of changes of gene modules involved in carbohydrate transport and metabolism, Ca^2+^ signaling transduction, nitrogen metabolism and oxidative phosphorylation. Among these genes, *NtTPS* and *NtGBE* are key genes in inhibiting PVY infection regulated by Mg. This may be related to the inhibition of trehalose metabolism in plants, which may affect the expression of PVY virulence related genes. *NtNR* and *NtPPase* are involved in nitrogen metabolism and energy metabolism in plants to maintain normal life activities, which are related to plant resistance to PVY infection. Our study also elucidates that Mg may induce the HR in plants by regulating Ca^2+^ signaling transduction, thereby inducing PVY resistance in tobacco. The results of VIGS assays suggest that *NbCML36* is not associated with PVY infection, which may be due to genetic redundancy. This study provides candidate genes for tobacco disease resistance breeding, and lays the foundation of future molecular mechanism interpretation of tobacco resistance against PVY infection regulated by Mg.

## Data availability statement

The datasets presented in this study can be found in online repositories. The names of the repository/repositories and accession number(s) can be found at: https://www.ncbi.nlm.nih.gov/genbank/, PRJNA903693.

## Author contributions

ZW and YW conceived the research project. RL and Xinyu Lan completed element spray tests. CX and ZW completed transcriptome sequencing tests. HG, FW, LJ, Xiao Lei, and MZ performed transcriptome data analysis and gene function validation. HG wrote the original draft. ZX revised the manuscript. ZW and YW edited the final manuscript. All authors contributed to the article and approved the submitted version.

## Funding

This research was funded by the Planning and Management Project of Sichuan Company for controlling plant vector-borne viruses, grant number SCYC202214 and SCYC202311.

## Conflict of interest

The authors declare that the research was conducted in the absence of any commercial or financial relationships that could be construed as a potential conflict of interest.

## Publisher’s note

All claims expressed in this article are solely those of the authors and do not necessarily represent those of their affiliated organizations, or those of the publisher, the editors and the reviewers. Any product that may be evaluated in this article, or claim that may be made by its manufacturer, is not guaranteed or endorsed by the publisher.

## Footnotes

^1^https://solgenomics.net/organism/Nicotiana_tabacum/genome
