# Supplementary material for: Transcriptome sequencing and functional verification revealed the roles of exogenous magnesium in tobacco anti-PVY infection
Source: Front Microbiol. 2023 Jul 27;14:1232279. doi: 10.3389/fmicb.2023.1232279 (PMC10414187; doi:10.3389/fmicb.2023.1232279)
Supplement: Supplementary file 9 [file Data_Sheet_1.docx]

Supplementary Material

## Supplementary Figures





**Supplemental Figure 1. Correlation heatmap of these four treatments with three biological replicates.** The closer Pearson's correlation coefficient (R) is to 1, the stronger the correlation between the two duplicate samples is.
